# Introduction and effect of natural selection analysis at common mutations of SARS-CoV-2 spike gene in Iran

**DOI:** 10.1016/j.virusres.2023.199202

**Published:** 2023-09-01

**Authors:** Fatemeh Nedaei, Ahmad Reza Esmaeili Rastaghi, Esmaeil Goodarzi, Hoora Haji Mullah Asadullah, Fatemeh Mirhadi, Abolfazl Fateh

**Affiliations:** a Department of Mycobacteriology and Pulmonary Research, Pasteur Institute of Iran, Tehran, Iran; b Department of Parasitology, Pasteur Institute of Iran, Tehran, Iran; c Department of Biology, Science and Research Branch, Islamic Azad University, Tehran, Iran; d Faculty of Medicine, Alborz University of Medical Sciences, Alborz, Iran; e Department of Medical science, Science and Research Branch, Islamic Azad University, Tehran, Iran; f Microbiology Research Center (MRC), Pasteur Institute of Iran, Tehran, Iran

**Keywords:** COVID-19, SARS-CoV-2, Spike protein, Haplotype

## Abstract

The epidemic of coronavirus disease 2019 (COVID-19) was caused by the severe acute respiratory syndrome coronavirus 2 (SARS-CoV-2). The spike (S) protein of SARS-Cov-2 is composed of two subunits, S1 and S2. This study aimed to describe SARS-CoV-2 haplotypes in Iranians based on the S gene, which plays a key role in the receptor recognition and cell membrane fusion proses. 95 positive saliva samples for SARS-CoV-2 were amplified and sequenced for the S gene. The sequences were classified into 35 haplotypes, which 11 haplotypes were new (H1, H2, H3, H4, H6, H7, H11, H13, H15, H16, H25) and have not been reported so far. Amino acid substitutions were found at 40 positions that 23 were located at S1 subunit and 16 were at S2 subunit and one was at cleavage loop (P681H/R), thus polymorphisms at S1 subunit were found to be higher than S2. The neutrality index (NI) analyses showed a negative departure from the neutral substitution patterns (NI > 1) for S1 and S2 subunit in the studied sequences. The co-occurrence of B-cell epitopes and mutation sites were found in seven positions with more probably to be exposed the immune system pressure. In conclusion, the results provide the significant data to design an effective vaccine based on this protein.

## Highlights

• The sequences were classified into 35 haplotypes, which 11haplotypes were new (H1, H2, H3, H4, H6, H7, H11, H13 H15, H16, H25) and have not been reported so far.
• Amino acid substitutions were found at 40 positions that 23 were located at S1 subunit and 16 were at S2 subunit and one was at cleavage loop.
• The neutrality index (NI) analyses showed a negative departure from the neutral substitution patterns (NI>1) for S1 and S2 subunit in the studied sequences.

## 1 Introduction

The pandemic of coronavirus disease 2019 (COVID-19) was caused by SARS-CoV-2 (Zhu et al., 2020). Globally, as of 3April 2023 over 765 million confirmed cases and over 6.9 million deaths have been reported (World Health Organization, 2023). SARS-CoV-2 is a single-strand RNA-enveloped virus. The genome size is 29,881 bp in length encoding structural proteins including the S, E, M, N genes and nonstructural proteins such as 3-chymotrypsin-like protease, papain-like protease and RNA-dependent RNA polymerase (Chan et al., 2020; Goldsmith et al., 2020; Lu et al., 2020).

The surface of the virus is covered by a large number of glycosylated spike proteins which bind to the host cell receptor angiotensin-covering enzyme 2 (ACE2) mediating viral cell entry (Letko et al., 2020). Among all human coronaviruses, the S protein is highly conserved and critical to the virus life cycle (Huang et al., 2020).

The SARS-CoV-2 S protein is made of 1273 amino acid, which is composed of two subunits (S1 and S2) (Huang et al., 2020). The S1 subunit includes an N-terminal domain and the receptor binding domain (RBD) and the S2 subunit contains an internal membrane fusion peptide, two heptapeptide repeat sequences (HR1 and HR2), a membrane-proximal external region and a trans membrane domain (TM) (Robson, 2020).

Evidences show that the S protein is the main surface glycoprotein and the most relevant source of antigens for vaccine development (Martinez-Flores et al., 2021; Sharma et al., 2020). On the other hand, the emergence of new variants may be occurred by random mutations, co-infection with different strains and recombination between their genomes that may increase their transmissibility and/or virulence with a possible reduction of vaccine effectiveness (Gui et al., 2017; Yu et al., 2017). Although antigenic variation is one of the major problems to develop an effective anti-COVID-19 vaccine, Knowledge of the virus genome polymorphisms, help us to design an effective vaccine.

To date, five SARS-CoV-2 variants including Alpha, Beta, Gamma, Delta, and Omicron variants have been declared variants of concern (VOCs) by the World Health Organization on the basis that they exhibit substantially altered transmissibility or immune escape, warranting close monitoring. Compared to the original virus, VOCs have increased transmissibility and have the potential for increasing disease severity (Carabelli et al., 2023; Choi and Smith, 2021).

Early investigations classified available SARS-CoV-2 genome sequences from all over the world into haplotype groups (Forster et al., 2020; Pachetti et al., 2020; Tang et al., 2020). A haplotype includes a set of variants along a chromosome that can be limited to a single gene or include multiple genes (Leitwein et al., 2020). An investigation has grouped the SARS-CoV-2 genome sequences, pertaining to viruses isolated from patients prior to the end of March 2020 into 66 haplotypes (Safari et al., 2021). Other studies have labeled haplotypes as clades, cluster, or lineages (Liu et al., 2020; Rambaut et al., 2020). Here, the SARS-CoV-2 S gene sequences obtained from samples, collecting from different regions of Iran, were classified into haplotypes based on the S gene by using Dna SP Software.

## 2 Material and methods

### 2.1 Sample collection

**Fig. 1:**
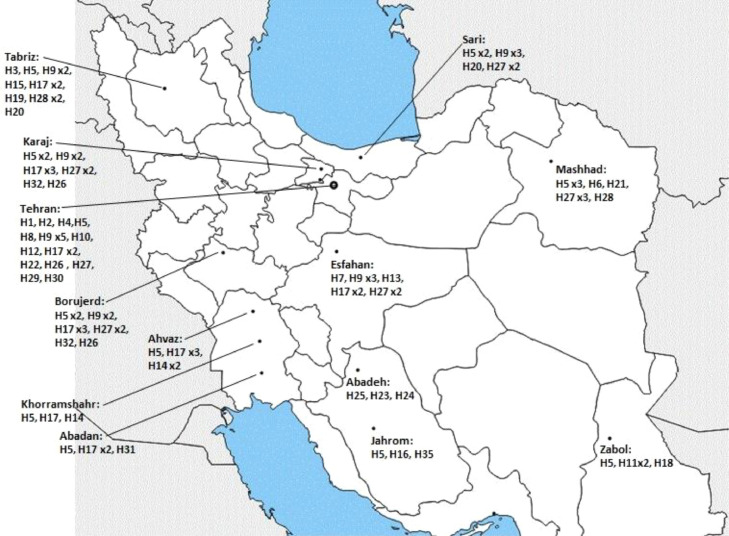
Locations of sample collection and haplotype distribution of 95 SARS-CoV-2 samples in Iran.

A total of 95 positive saliva samples were collected from 14 different cities including Tehran, Karaj, Sari, Tabriz, Mashhad, Hamedan, Boroujerd, Esfahan, Zabol, Ahvaz, Khorramshahr, Abadan, Abadeh, Jahrom in Iran during 2020˗ 2023 (Fig. 1). The samples were obtained from patients with symptomatic COVID-19 infections who had referred to medical centers. All studied individuals gave informed consent according to Universidad ethical guidelines. This study has the ethical approval of the Islamic Azad University Sari Branch (IR.IAU.SARI.REC.1402.111).

The characteristics of the studied individuals and their travel history were recorded before sampling. The majority of the patients (∼63%) were between 40 and 60 years old. Saliva samples were self-collected under the observation of a healthcare worker. Viral agents in specimen aliquots were inactivated in guanidinium-thiocyanate-based lysis buffer at room temperature for 10 min before extraction of viral nucleic acid.

### 2.2 RNA extraction, cDNA synthesis, and PCR

RNA was extracted from clinical samples with the High pure viral RNA kit (Roche) according to the manufacturer's instructions. After RNA extraction, cDNA was synthesized by cDNA synthesis Kit (Yektatajhiz, Iran). Overlapping PCR was performed to amplify the S gene and primers were designed as described in Salles et al. (2022). PCR amplifications were performed in a 25 μl PCR reaction volume containing 0.2 μM of each primer pair, 1.5 mM MgCl2, 1 × PCR buffer (50 mM KCl, 10 mM Tris–HCl, pH 8.3), 0.2 mM dNTP and 0.5 u of Taq DNA polymerase (Bioron, Ludwigshafen, Germany). The cycling parameters to amplify the fragments were as follows: initial denaturation at 94 °C for 10 min, 35 cycle of denaturation at 94 °C for 1 min, annealing at 57 °C for 1 min, extension at 72 °C for 1 min and a final extension at 72 °C for 10 min (Applied bio system, Hong Kong, China).

### 2.3 Nucleotide sequencing and data analysis

PCR products were sequenced using ABI Big Dye Terminator Reaction ready kit (PE Applied Bio system, Foster City, USA) and an automated DNA sequencer (ABI 310 Genetic Analyzer; PE Applied Bio system). Nucleotide sequences were aligned using MEGA7 (ver.7.0.26). The S1 sequence of the wuhan-Hu-1 (accession No. NC045512) isolate was used as the reference sequence. The haplotypes were classified based on the S nucleotide sequence using Dna SP (ver.5.10.01). The haplotype diversity (Hd), polymorphic or segregating sits (S), nucleotide diversity (π) and average number of pairwise nucleotide differences within the population (K) were estimated using Dna SP program. Evidence of recombination including Recombination between adjacent polymorphic site (Rb), Recombination per gene (Ra) and minimum number of recombination events (Rm) was also measured using Dna SP program.

To test the signature of neural selection under the null hypothesis based on a comparison of substitutions within and between sequences, the MC Donald and Kreitman test was performed with a single Middle East respiratory syndrome (MERS)-related coronavirus isolate HCoV-EMC (accession No. NC019843) as an out group, under neutrality the ratio of non-synonymous (Pn) to synonymous (Ps) mutations within species is expected to be equal to that of non-synonymous (Dn) to synonymous (Ds) mutations among species. However, under positive or negative selection these ratio will not be equal (Neutrality Index; NI = 1). The higher value of fixed differences among species to that with in species for non-synonymous mutations [Dn/Pn>Ds/Ps] indicates that genetic polymorphisms have been subjected to positive selection. To determine the significant deviations from neutrality, the two tailed Fisher's exact test was computed (*P* > 0.05). Also, Tajima's test and other analysis were performed using Dna SP software to study the effects of natural selection in the population including Fu and Li's D* and F* tests (Fu and Li, 1993; Tajima, 1989).

Phylogenetic tree based on the S gene sequences was performed by the neighbor joining method in MEGA software to analyses the genetic relationships among the haplotypes from this study and previously reported SARS-CoV-2 sequences from different geographic regions.

Fixation-statistics (Fst) was performed to measure the extent of allele diversity among populations from Germany, Switzerland, Colombia, and USA using Dna SP program. The Fst interpretation at each locus is based on four categories as follows: no genetic differentiation (0), low genetic differentiation (0–0.05), moderate differentiation (0.05–0.15), a value between 0.15–0.25 indicates high differentiation and above 0.25 shows very high genetic differentiation (Balloux and Lugon-Moulin, 2002).

The ABCpred server (http://www.imtech.res.in/raghava/abcp) was used to predict the potential B-cell epitopes (Saha and Raghava, 2006). Finally, a three-dimensional structure of SARS-CoV-2 S antigen (isolate Wuhan-Hu-1; Gen Bank accession No. MN908947) was generated to show the distribution of polymorphic sites using Weblab Viewer Lite 4.2.

## 3 Results

### 3.1 Genetic variation and natural selection

The fragments of the S gene were successfully amplified and sequenced from 95 SARS- CoV-2 infected Saliva samples. The nucleotide sequence data in this study are available in the Gen Bank database under accession numbers OR121421—OR121455. The 95 sequences were classified into 35 haplotypes where haplotype H5 was predominant (17.9%, *n* = 17). The H9 and H17 were observed in 15 isolates each (15.8%, *n* = 15) and H27 in 10 isolates (Table 1). Amino acid sequence polymorphisms of the 35 haplotypes, reported in this study, are available in Table 2. Also, the Gen Bank BLAST searches for each haplotype showed that 11 haplotypes were novel (H1, H2, H3, H4, H6, H7, H11, H13 H15, H16, and H25) and have not been reported so far.

**Table 1:** Distribution of COVID-19 cases and haplotype frequency by different age categories.

Age Groups	Frequency (%)	Haplotypes (H)
>10	2.1	H9, H17
10–20	4.2	H5, H9 (x2), H17
20–30	7.4	H5 (x2), H9, H17 (x2),H27
30–40	12.6	H4, H5 (x2), H9 (x4), H17 (x2),H18, H28, H29, H27
40–50	26.3	H1,H3, H5 (x4), H8, H9 (x2), H11, H14, H15, H17(x3), H21, H24, H25, H26, H27 (x3), H30, H31, H35
50–60	36.8	H2,H5 (x4), H6, H7, H9 (x3), H10, H11, H12, H13, H14 (x2),H16,H17 (x3),H19, H20 (x2), H22, H23, H26, H27 (x5), H28 (x2), H32, H33,
60–70	7.4	H5 (x3), H9, H17 (x2), H34
70 >	3.2	H5, H9, H17
Total (%)	100

**Table 2:** Amino acid polymorphisms in the SARS-CoV-2 spike protein among Iranian (*H* = 35) and their comparison with Wuhan-Hu-1 sequence.

	18	19	20	26	54	80	95	138	142	153	178	190	215	417	452	477	478	484	501	558	570	614	655	675	681	701	702	716	822	829	832	850	899	931	950	982	1027	1118	1176	1237	1247
Wuhan	L	T	T	P	L	D	T	N	G	M	D	R	D	K	L	S	T	E	N	K	A	D	H	Q	P	A	E	T	L	A	G	I	A	I	D	S	T	D	V	M	C
H1	L	T	T	P	L	D	T	D	G	M	D	R	D	K	L	S	T	E	Y	K	D	G	H	Q	H	A	E	I	L	A	G	I	A	I	D	A	T	H	V	V	C
H2	L	T	T	P	L	A	T	D	G	M	D	R	G	N	L	S	T	E	Y	K	A	G	H	Q	P	V	E	T	L	A	G	I	A	V	D	S	T	D	V	M	C
H3	F	T	N	S	L	D	T	Y	G	M	D	R	G	T	L	S	T	K	Y	R	A	G	Y	Q	P	A	E	T	L	A	G	I	A	I	D	S	I	D	F	M	C
H4	L	R	T	P	L	D	I	D	D	M	D	R	D	K	R	S	K	E	N	K	A	G	H	Q	R	A	V	T	L	A	G	I	A	I	N	S	T	D	V	M	F
H5	L	T	T	P	L	D	T	D	G	M	D	R	D	K	L	S	T	E	Y	K	D	G	H	Q	H	A	E	I	L	A	G	I	A	I	D	A	T	H	V	M	C
H6	F	T	T	P	L	A	T	D	G	M	D	R	G	N	L	S	T	K	Y	K	A	G	H	H	P	V	E	T	L	A	G	I	A	I	D	S	T	D	V	M	C
H7	F	T	T	P	L	A	T	D	G	M	D	R	G	K	L	S	T	K	Y	K	A	G	H	H	P	V	E	T	F	A	G	I	A	I	D	S	T	D	V	M	C
H8	F	T	T	P	L	A	T	D	G	M	D	R	G	N	L	S	T	K	Y	K	A	G	H	Q	P	V	E	T	L	A	G	I	A	I	D	S	T	D	V	M	C
H9	L	T	T	P	L	D	T	D	G	M	D	R	D	K	L	S	T	E	Y	K	A	G	H	Q	P	A	E	T	L	A	G	I	A	I	D	S	T	D	V	M	C
H10	L	T	T	P	L	D	T	D	G	M	D	R	D	K	L	S	T	E	N	K	A	G	H	Q	P	A	E	T	L	A	G	I	A	I	D	S	T	D	V	M	C
H11	F	T	T	P	F	A	T	D	G	M	D	R	G	N	L	S	T	K	Y	K	A	G	H	Q	P	V	E	T	L	A	G	I	A	I	D	S	T	D	V	M	C
H12	L	T	T	P	L	D	T	D	G	M	D	R	D	K	L	S	T	E	N	K	A	G	H	Q	P	A	E	T	L	A	G	I	A	I	D	S	T	D	V	M	C
H13	F	T	T	P	L	A	T	D	G	M	D	R	G	K	L	S	T	E	Y	K	A	G	H	Q	P	V	E	T	L	A	G	I	S	I	D	S	T	D	V	M	C
H14	L	T	T	P	L	D	T	D	G	M	D	R	D	K	L	S	T	E	N	K	A	G	H	Q	P	V	E	T	L	A	G	I	A	I	D	S	T	D	V	M	C
H15	F	T	N	S	L	D	T	D	G	M	D	S	D	K	L	S	T	K	Y	R	A	G	Y	Q	P	A	E	T	L	A	G	I	A	I	D	S	I	D	F	M	C
H16	F	T	N	S	L	D	T	Y	G	M	D	S	D	K	L	S	T	K	Y	R	A	G	Y	Q	P	A	E	T	L	A	G	I	A	I	D	S	I	D	F	M	C
H17	F	T	N	S	L	D	T	Y	G	M	D	R	D	T	L	S	T	K	Y	K	A	G	Y	Q	P	A	E	T	L	A	G	I	A	I	D	S	I	D	F	M	C
H18	F	T	N	S	L	D	T	Y	G	M	D	S	D	T	L	S	T	K	Y	R	A	G	Y	Q	P	A	E	T	L	A	G	I	A	I	D	S	I	D	F	M	C
H19	F	T	N	S	L	D	T	Y	G	M	D	S	D	T	L	S	T	K	Y	R	A	G	Y	Q	P	A	E	T	L	A	G	I	A	I	D	S	I	D	F	M	C
H20	F	T	N	S	L	D	T	D	G	M	D	S	D	T	L	S	T	K	Y	R	A	G	Y	Q	P	A	E	T	L	A	G	I	A	I	D	S	I	D	F	M	C
H21	F	T	N	S	L	D	T	Y	G	T	D	S	D	T	L	S	T	K	Y	R	A	G	Y	Q	P	A	E	T	L	A	G	I	A	I	D	S	I	D	F	M	C
H22	F	T	N	S	L	D	T	Y	G	M	N	S	D	T	L	S	T	K	Y	R	A	G	Y	Q	P	A	E	T	L	A	G	I	A	I	D	S	I	D	F	M	C
H23	F	T	N	S	L	D	T	Y	G	M	D	S	D	T	L	S	T	K	Y	K	A	G	Y	Q	P	A	E	T	L	A	G	I	A	I	D	S	I	D	F	M	C
H24	F	T	N	S	L	D	T	Y	G	M	D	S	D	T	L	S	T	K	Y	R	A	G	Y	Q	P	A	E	T	L	A	D	I	A	I	D	S	I	D	F	M	C
H25	L	R	T	P	L	D	I	D	D	M	D	R	D	K	R	S	K	E	N	K	A	G	H	Q	R	A	E	T	L	A	G	I	A	I	D	S	T	D	V	M	C
H26	L	R	T	P	L	D	I	D	D	M	D	R	D	K	R	S	K	E	N	K	A	G	H	Q	R	A	E	T	L	A	G	I	A	I	N	S	T	D	V	M	C
H27	L	R	T	P	L	D	T	D	D	M	D	R	D	K	R	S	K	E	N	K	A	G	H	Q	R	A	E	T	L	A	G	I	A	I	N	S	T	D	V	M	C
H28	L	R	T	P	L	D	I	D	D	M	D	R	D	K	R	S	K	E	N	K	A	G	H	Q	R	A	E	T	L	A	G	I	A	I	N	S	T	D	V	M	C
H29	L	R	T	P	L	D	I	D	D	M	D	R	D	K	R	S	K	E	N	K	A	G	H	Q	R	A	E	T	L	A	G	I	A	I	N	S	T	D	V	M	C
H30	L	R	T	P	L	D	I	D	D	M	D	R	D	K	R	S	K	E	N	K	A	G	H	Q	R	A	E	T	L	T	G	I	A	I	N	S	T	D	V	M	C
H31	L	R	T	P	L	D	I	D	D	M	D	R	D	K	R	S	K	E	N	K	A	G	H	Q	R	A	E	T	L	A	G	L	A	I	N	S	T	D	V	M	C
H32	L	R	T	P	L	D	I	D	D	M	D	R	D	K	R	S	K	E	N	K	A	G	H	Q	R	A	E	T	L	A	G	I	A	I	N	S	T	D	V	M	C
H33	L	R	T	P	L	D	I	D	G	M	D	R	D	K	R	S	K	E	N	K	A	G	H	Q	R	A	E	T	L	A	G	I	A	I	N	S	T	D	V	M	C
H34	L	R	T	P	L	D	I	D	D	M	D	R	D	K	R	I	K	E	N	K	A	G	H	Q	R	A	E	T	L	A	G	I	A	I	N	S	T	D	V	M	C
H35	L	R	T	P	L	D	I	D	D	M	D	R	D	K	R	S	K	E	N	K	A	G	H	Q	R	A	E	T	L	A	G	I	A	I	N	S	T	D	V	M	C

Analysis and comparison of the sequences with the isolate Wuhan-Hu-1 at the nucleotide level showed that point mutations occur at 50 sites (19 singleton variables and 31 parsimony informative sites).

Computing the McDonald and Kreitman test to determine whether natural selection contributes to the diversity within and between species showed an excess of synonymous substitutions between species and nonsynonymous substitutions within species compared to the neutral substitutions (PnDs/PsDn > 1) (Table 3). Neutrality index was calculated to be 2.110, 1.712 and 1.509 for the S1, S2+cleavage site and the entire S gene, respectively indicating an excess of polymorphic variation compared to the neutral substitutions due to negative selection and statistically nonsignificant (Fisher' s exact text *P* < 0.05) (Table 3). Also, the values of D* (F&L) and F* (F&L) tests (Table 5) showed a negative departure from neutral mutations at S1, S2+cleavage site and full length of S gene sequences in the studied population.

**Table 3:** Null hypothesis results with the McDonald and Kreitman test.

S-protein of SARS-CoV-2	polymorphic changes within SARS-CoV-2		Polymorphic changes between species		NI	Fisher's exact test
	Pn	Ps	Dn	Ds		
S1-Subunit	20	4	0.183	2.110	661	279
S2 Subunit + S1/S2 cleavage site	16	7	0.296	1.712	547	278
Total	36	11	0.267	1.509	1208	557

Ps – the number of synonymous substitutions within the spike gene; Pn – the number of non-synonymous substitutions within the spike gene; Ds – the number of synonymous changes between species; Dn – the number of non-synonymous changes between species; NI – neutrality index; The values were not significant with *P* < 0.05.

Amino acid changes were found at 40 sites where 23 polymorphic sites located at S1 subunit and 16 sites were at S2 and one was at cleavage loop. Out of these polymorphic sites, 38 sites were dimorphic and two were trimorphic (K417T/N, P681H/R) (Table 4)

**Table 4:** Amino acid substitutions at spike protein of SARS-CoV-2 among 95 Iranian samples.

SARS-CoV-2 Spike protein (*n* = 95)					
S1 subunit			S2 subunit		
Codon	Mutations	Frequency	Codon	Mutations	Frequency
18	L/F	63/32	701	A/V	85/10
19	R/T	24/71	702	E/V	94/1
20	T/N	69/26	716	I/T	18/77
26	P/S	69/26	822	L/F	94/1
54	L/F	93/2	829	A/T	94/1
80	D/A	88/7	832	G/D	94/1
95	T/I	81/14	850	I/L	94/1
138	D/Y	72/23	899	A/S	94/1
142	D/G	24/71	931	I/V	94/1
153	M/T	94/1	950	D/N	72/23
178	D/N	94/1	982	S/A	77/18
190	R/S	85/10	1027	T/I	69/26
215	D/G	87/8	1118	D/H	77/18
417	N/K/T	5/66/24	1176	V/F	69/26
452	R/L	24/71	1237	M/V	94/1
477	S/I	94/1	1247	C/F	94/1
478	T/K	71/24	Cleavage site		
484	K/E	31/64	Codon	Mutations	Frequency
501	Y/N	66/29	681	H/P/R	18/53/24
558	K/R	85/10			
570	D/A	77/18			
655	H/Y	69/26			
675	Q/H	93/2			

Nucleotide sequence analysis was performed for S1, S2+cleavage site and overall S gene within 95 sequences. Total number of mutation sites for these regions were 27, 26 and 53, respectively (Table 5). The overall nucleotide diversity (π) and Hd for complete S gene were 0.0025 and 0.91, respectively. Also, the average number of pairwise nucleotide differences (K) was 9.822 (Table 5). Results indicated that the extent of genetic polymorphisms (Hd and π) was higher at S1 subunit than S2 (Table 5).

**Table 5:** DNA sequence polymorphisms estimation and neutrality tests at spike protein of SARS-CoV-2 among 95 Iranian samples.

	S	Singleton variable site	Parsimony informative site	Total no. of mutation	K	π	Hd ± SD	Tajima's D	D*(F&L)	F*(F&L)
S1 subunit	26	6	20	27	6.345	0.0031	0.898 ± 0.014	0.7561	−0.2396	−0.1078
S2 + S1/S2 cleavage site	24	13	11	26	3.476	0.0019	0.877 ± 0.014	−0.7687	−2.8216*	−2.882*
Total	50	19	31	53	9.822	0.0025	0.910 ± 0.015	0.0219	−1.9963	−1.5026

S – number of polymorphic (segregating) sites; K – average number of pairwise nucleotide differences; π – observed average pairwise nucleotide diversity; Hd – haplotype diversity; dN - dS – the difference between the rate of non-synonymous and synonymous mutations; D*(F&L) – Fu and Li's D* value; F*(F&L) – Fu and Li's F* value; * significant values with *P* < 0.05.

The Fst estimation at the S gene among sequences from Iran, Germany, Switzerland, Colombia and USA showed no genetic differentiation between populations from USA and Germany (Fst = 0.00) whereas the fixation index among isolates from other countries indicated very high genetic differentiation (Fst > 0.25) (Table 6).

**Table 6:** Genetic differentiation estimation at SARS-CoV-2 spike gene among populations from various geographic areas.

Country	Iran (*n* = 95)	Germany (*n* = 46)	Switzerland (*n* = 45)	Colombia (*n* = 14)	USA (*n* = 30)
Iran		0.0011*	0.0004*	0.0005*	0.0008*
Germany	0.4668		0.0015*	0.0016*	0.0000*
Switzerland	0.2699	0.9895		0.0001*	0.0012*
Colombia	0.2799	0.9448	0.6003		0.0012*
USA	0.3712	0.0000	0.8469	0.8119	

Fst values are showed in the lower left and *P*-values are showed in the upper right of the table; *n* – number of samples analyzed;* the Fst values were found as significant difference (*P* < 0.05).

**Fig. 2:**
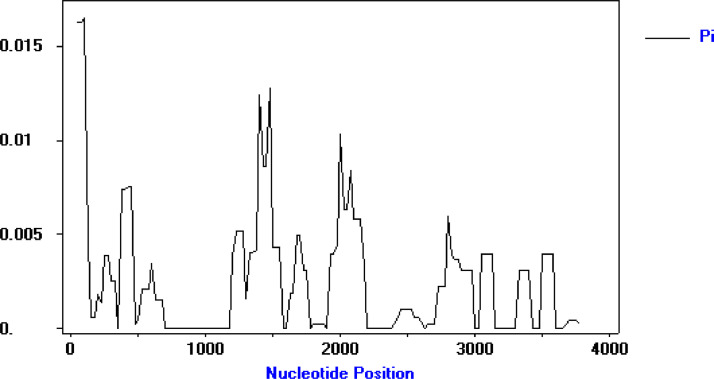
Sliding window plot of nucleotide diversity per site (π) at the spike gene in 95 Iranian samples with the window length 100 bp and step size of 25 bp. The analysis was performed using DnaSP ver. 5. 10. 01. The level of nucleotide diversity was high between the nucleotide positions 1 - 150 and then 1426–1525 at S1 subunit also nucleotide positions 2751–2850 at S2 subunit.

Analysis with sliding window plot (window length of 100- bp, step size of 25- bp) of the Dna SP package showed π diversity ranging from 0 to 0.1658. Maximum diversity was found between nucleotide positions 1–150 and then 1426–1525 at S1 subunit. The nucleotide positions 2751–2850 have also a high level of polymorphism at S2 subunit (Fig. 2).

Minimum number of recombination events between adjacent polymorphic sites (Rm) for the entire S gene was 6, whereas recombination between adjacent site and recombination per gene were 0.003 and 11.4, respectively.

### 3.2 Phylogenetic analysis

**Fig. 3:**
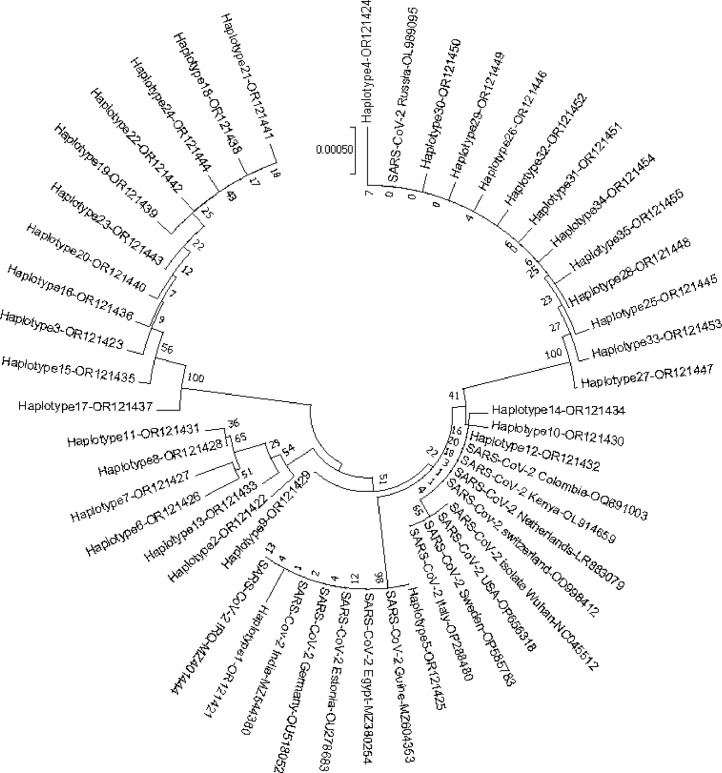
Phylogenetic tree based on the complete S gene sequences. The evolutionary history was reconstructed using the Neighbor-Joining method in MEGA7. The bootstrap consensus tree was inferred from 1000 replicates and values for the nodes are shown in the tree.

Phylogenetic analysis based on the S gene of SARS-CoV-2 put the isolates from different geographic regions of the world in different subdivision of phylogenetic tree (Fig. 3).

### 3.3 Analysis of the B-cell epitopes and molecule structure of SARS-CoV-2 S antigen

**Fig. 4:**
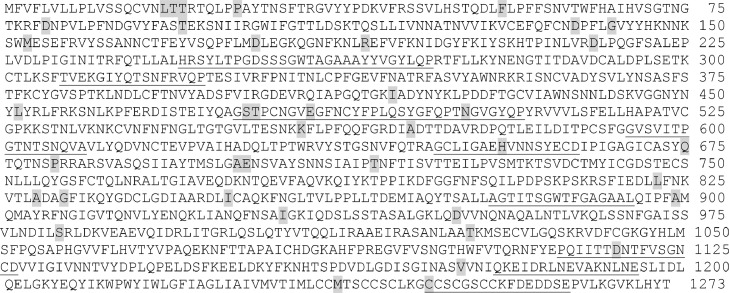
Location of detected SNPs in the present study in the predicted B-cell epitopes using ABCpred server. Seven mutation sites were found in the B-cell epitopes. Gray shadow: SNPs in the current study; underlined shows B-cell epitopes.

**Fig. 5:**
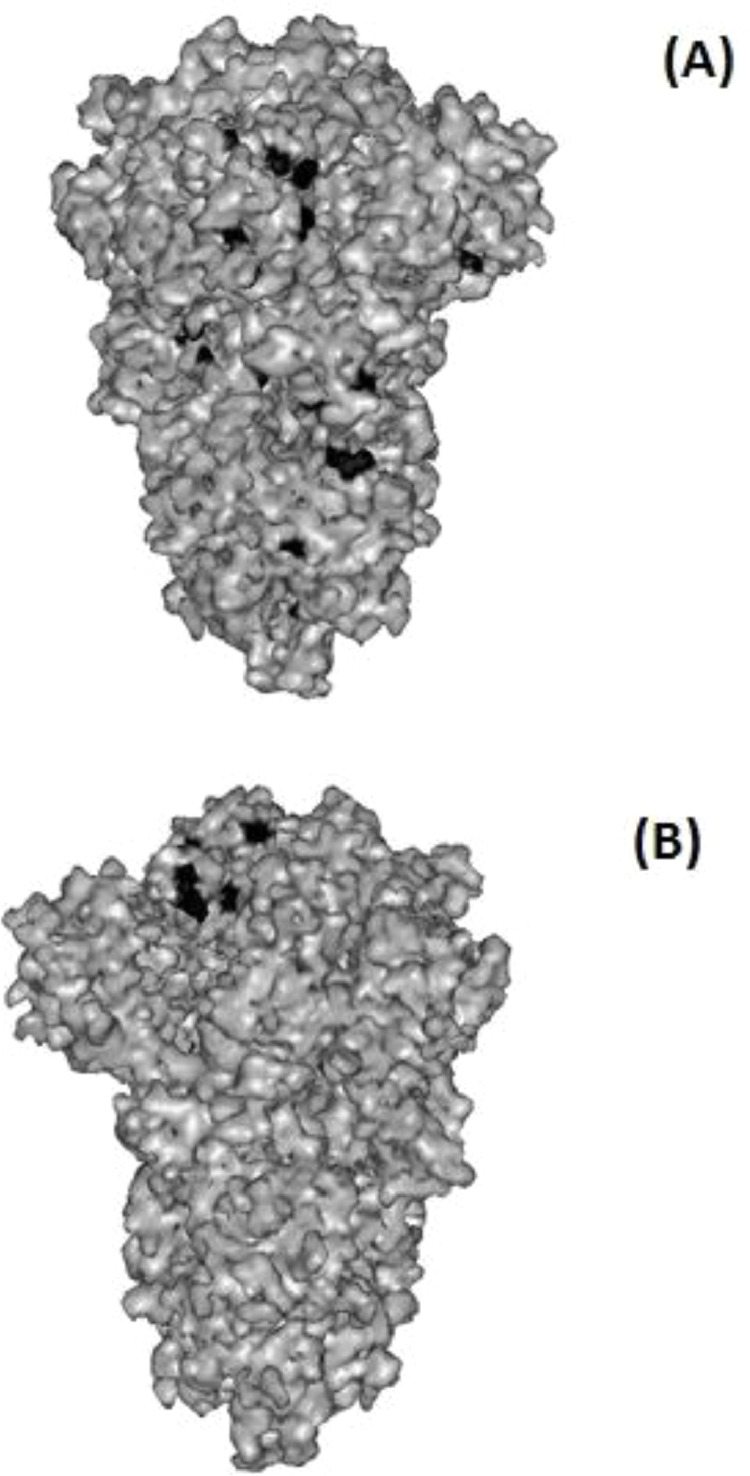
Crystal structure of Spike protein was generated with Web Lab Viewer Lite 4.2. The polymorphic sites are indicated in Black color. All other positions show invariant amino acids. Panels A and B are two opposite faces of the same structure.

The B cell epitopes predicted using ABCpred server. 172 residues were predicted by the threshold of 0.9 to show a given region as a reliable linear B-cell epitope (Fig. 4). The molecule structure of SARS-CoV-2 S antigen was constructed to determine the distribution of mutation sites. The results showed that almost all of these mutations were located in one side of the molecule (Fig. 5).

## 4 Discussion

This study was aimed to investigate the extent of genetic variations and effect of natural selection at SARS-CoV-2 spike protein among Iranian isolates. Although, antigenic polymorphism is a major problem to design an effective vaccine, the detailed knowledge of the virus antigenic variations is a prerequisite to design an effective vaccine in various regions of the world.

Haplotype distribution analysis of 95 sequences using Dna SP program result in 35 haplotypes among Iranian populations. In order to compare these haplotypes with the previously reported SARS-CoV-2 spike gene sequences in the GenBank database the BLAST search was performed, based on the results 11 haplotypes were novel (H1, H2, H3, H4, H6, H7, H11, H13, H15, H16, H25) and rest of them previously identified among isolates from Iran (H8, H10, H17, H18, H19, H20, H21, H22, H34), India (H5), Iraq (H5), Russia (H28), Switzerland (H5, H8, H31), Wales (H9), Denmark (H28, H26), Brazil (H29), England (H12, H33), Malaysia (H14), Germany (H23, H27, H32), USA (H24, H30), Mexico (H35). Some haplotypes were obtained from individuals with a travel history to India (H5) and Russian (H28) before sampling, it is suggested that migration of human hosts from/to neighboring countries is responsible for the existence of the similar variants among these regions.

K417N/T, L452R, E484K, N501Y, P681H, P681R mutations reported in this study was classified in Alpha-Delta variants. K417N appears in the Beta and Omicron variants, while K417T appears in Gamma (Hadj Hassine, 2022). Both of them are resistant to neutralizing antibodies (Khan et al., 2021). K417N mutation in Omicron variant reduces protein stability and increases disease susceptibility (Kumar et al., 2022). L452R is found in the Delta variants and appears to enhance ACE2 receptor binding affinity and can diminish the interaction with vaccine-elicited antibodies (Hadj Hassine, 2022; Tchesnokova et al., 2021). E484K is shared by Beta and Gamma variants (Hadj Hassine, 2022). This mutation improves the capability to escape the immune system by affecting antibody recognition (Greaney et al., 2021). The substitution N501Y is shared by Alpha, Beta, Gamma, and Omicron variants (Hadj Hassine, 2022; Kumar et al., 2022). The N501Y mutation is one of 15 Omicron variant mutations found in RBD. Although it improves protein stability and is well tolerated; however, it is disease-prone (Kumar et al., 2022). This change induces higher concentrations in the pharynx and the nasal cavities and therefor, increases its transmission rate (Conti et al., 2021). The P681H and P681R substitutions are located at the cleavage site and have been detected in Alpha and Delta, respectively (Hadj Hassine, 2022). The P681R mutation increases viral fusogenicity (Liu et al., 2022).

Also, A701V mutation reported in this investigation is significantly resistant to neutralizing antibodies elicited by immunization (Wang et al., 2023), indicating reduced protective efficacy of the vaccines against these SARS‐CoV‐2 variants and enhance the infectivity of the virus, the evidence shows the necessity of vaccine renewal and providing direction for the development of new vaccines.

Analysis of nucleotide diversity showed more variation at S1 subunit than S2, in other words S2 subunit is more conserved across coronaviruses. This result is similar to previous studies (Ng et al., 2021; Premkumar et al., 2020; Shah et al., 2021). Furthermore a high level of polymorphism were found among Iranian isolates (*H* = 35) that may be occurred due to recombination between genomes of SARS CoV-2 strains with passage time. In other words, in addition to random mutations, Co-exiting viruses and recombination between their genomes may significantly lead to the emergence of new SARS-CoV-2 haplotypes (Taghizadeh et al., 2021).

The McDonald Kreitman test was performed as a test of neutrality and findings showed a negative departure from neutral substitution patterns (NI > 1) for S1, S2+cleavage site and the entire length of S gene in the studied sequences supported by the results of the D* (F&L) and F*(F&L) test indicating host immune selection pressure due to antibodies elicited by infection, vaccination or both. The result is in agreement with previous report by (Lu et al., 2023) whereas is in contrast to some other reports (Hou et al., 2022; Morales et al., 2021).

To design an effective vaccine against SARS-CoV-2, selection of antigenic epitopes that induce protective antibodies would be a conductive strategy. Indeed, the co-occurrence of B-cell epitopes and variable sites represents the regions which are more likely to be exposed to the immune system and such residues have been found in 7 positions in this study. Therefore, most of the mutation sites have located outside B-cell epitopes.

In conclusion, present study extends our knowledge of polymorphisms and the prevalence of spike protein alleles in natural populations of SARS-CoV-2 from Iran. Moreover, combination of this information with similar reports from other geographical regions can be helpful in developing vaccine constructs that will elicit immune response against all known variants of the virus.

## Funding

None.

## CRediT authorship contribution statement

**Fatemeh Nedaei:** Investigation, Methodology, Software, Data curation, Writing – original draft, Writing – review & editing, Methodology, Conceptualization, Supervision. **Ahmad Reza Esmaeili Rastaghi:** Investigation, Methodology, Software, Data curation, Writing – original draft, Writing – review & editing. **Esmaeil Goodarzi:** Investigation, Methodology, Software, Data curation, Writing – original draft, Writing – review & editing. **Hoora Haji Mullah Asadullah:** Methodology, Data curation, Writing – original draft, Writing – review & editing. **Fatemeh Mirhadi:** Methodology, Data curation, Writing – original draft, Writing – review & editing. **Abolfazl Fateh:** Conceptualization, Writing – review & editing, Supervision.

## Declaration of Competing Interest

The authors declare that they have no known competing financial interests or personal relationships that could have appeared to influence the work reported in this paper.

## Data Availability

Data will be made available on request.
